# Dynamic Hierarchical Self-Assemble Small Molecule Structure Hexabenzocoronene for the High-Performance Anodes Lithium Ion Storage

**DOI:** 10.1186/s11671-019-2903-4

**Published:** 2019-02-26

**Authors:** Dawei He, Fuyan Xiao, Zhou Wang, Aolin He, Ruijiang Liu, Guofan Jin

**Affiliations:** 1 Affiliated Kunshan Hospital, Jiangsu University, Kunshan, 215300 People’s Republic of China; 2 College of Vanadium and Titanium, Panzhihua University, Panzhihua, 617000 People’s Republic of China; 3 School of Pharmacy, Jiangsu University, Zhenjiang, 212013 People’s Republic of China

**Keywords:** Hexabenzocoronene, Dynamic hierarchical self-assemble, *d*-spacing, Rearrange

## Abstract

This study examined the characteristics of small molecular structure nano-graphene in a dynamic hierarchical self-assembly and found that graphene is rearranged under its own pressure during dynamic aggregation and water ripples are formed by the *d*-spacing. The composition and structure were studied using a range of material characterization techniques. No covalent bonds were observed between molecules, and the self-assembled driving force was the only intermolecular interaction: Van der Waals’ force in the intra-layer and π–π interactions between layers. The arranged-rearranged structures provided a range of lithium ion shuttle channels, including the space between layers and diffusing through the nanosheets, which decrease the diffusion distance of lithium ions remarkably and reduce the irreversible capacity of the battery.

**Electronic supplementary material:**

The online version of this article (10.1186/s11671-019-2903-4) contains supplementary material, which is available to authorized users.

## Introduction

The development of green alternative energy sources has received considerable interest. Recently, nano-graphene and graphene composites attracted interest for used as lithium ion anodes [1–3]. In addition, a variety of core–shell structures with carbonaceous materials encapsulated silicon or metal nanostructure have been proposed to alter the performance of the anode materials [4]. Furthermore, graphene is one of the most promising materials to replace graphite and has been studied widely since Professor Andre Konstantin Geim and Konstantin Sergeevich produced stable graphene in 2004 using the deceptively simple Scotch tape method [5, 6]. Other methods of producing graphene include liquid phase and thermal exfoliation [7–9], chemical vapor deposition [10, 11], and synthesis on SiC [12, 13]. Graphene has a hexagonal honeycomb lattice structure, and its amazing properties have stimulated strong interest [14–20].

**Fig. 1:**
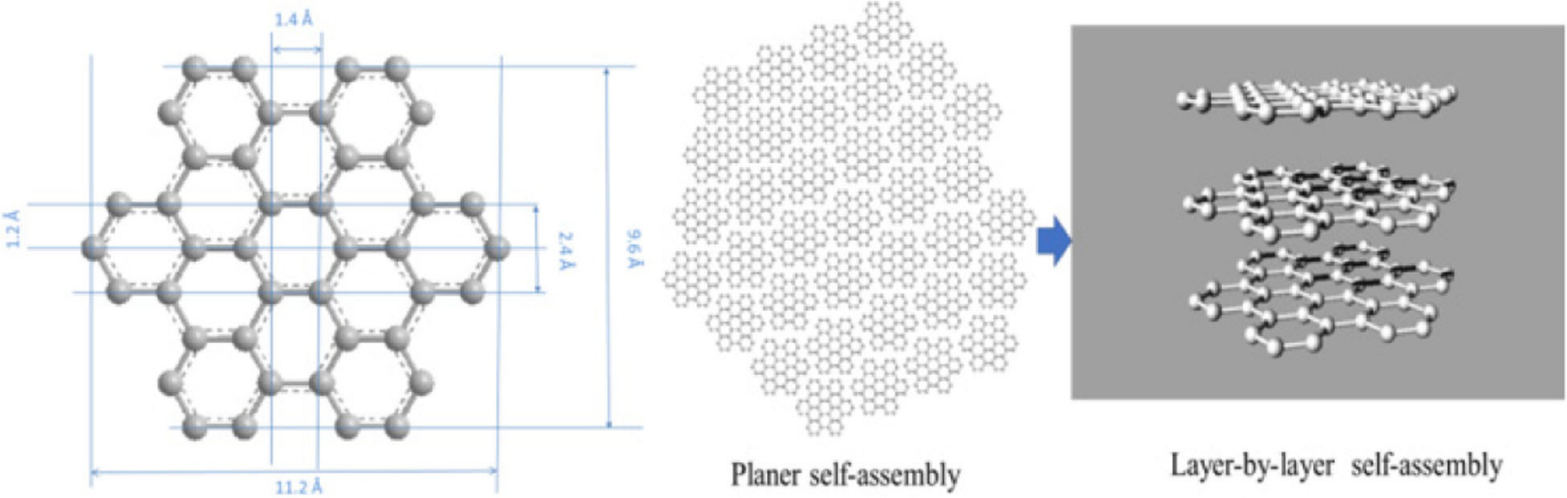
Hexabenzocoronene structure and self-assembly diagram

Hexabenzocoronene (HBC, hereafter) is a representative example of nano-graphene that has been well studied [21–30]. The smaller modular sizes and size-tunable are the main features. HBC is one of the allotropes of carbon with a layered structure of sp^2^ carbon atoms. Each layer has a hexagonal honeycomb structure called a nano-graphene sheet (Fig. 1) [31]. While the chemistry of nano-graphene has been well established, its ability to overlap and aggregate in a generalized nano-morphology molecule is not completely understood. Therefore, determining how nano-sized graphene molecules are stacked and how the stacked sheets interact is important.

This paper introduces the dynamic hierarchical self-assembly structure–function relationship of hexabenzocoronene. By observing the *d*-spacing generated via the dynamic self-assembly at the molecular level and the relationship between the clusters of nano-graphene, an in-depth analysis of the formation factors inside of nano-graphene was analyzed further.

## Methods/Experimental

### Materials

Hexabenzocoronene was synthesized according to a previously reported procedure [32–35]. All solvents were freshly distilled from proper dehydrating agents under argon gas. All chemicals are analytical grade and purchased from Shanghai Chemical Corp. Thin-layer chromatography (TLC) was performed on silica gel 60 F254 (Merck DGaA, Germany). The electrolyte solution was purchased from Shanghai Annaiji Technology Co., Ltd. The electrolyte solution is made up from 0.1 M tetra-*n*-butylammonium perchlorate (TBAP). Deionized water is used for all experiments.

### Characterization

The morphology and lattice fringe were observed using a scanning electron microscope (SEM, JEOL JCM-6000Plus), transmission electron microscope (TEM, JEOL H-7000), and high-resolution transmission electron microscope (HRTEM, JEOL JEM-2100).

### Electrochemical Measurements

Electrochemical measurements were performed on the Shanghai Chenhua CHI660e system. A three-electrode system is used, a platinum wire for the counter electrode, a platinum plate with a fixed of electrode, and a saturated calomel electrode for the reference electrode. The concentration of the supporting electrolyte TBAP was 0.1 mol/L, and the analytical pure solvent was acetonitrile (ACN). Firstly, polish the platinum carbon compound electrode vertically on the circular gauze on the glass brick (paint “8”, 0.05 μm aluminum powder and water as friction agent); secondly, rinse off the white aluminum with distiller water and then use ultrasonic for 1 min by acetone; and finally, use ear ball washed and blown dry. Then, the suspension of the hexabenzocoronene sample was dropped on the surface of the glassy carbon compound electrode, and the solvent was naturally evaporated to dryness. Then 0.1 M tetra-*n*-butylammonium perchlorate and 0.1 mM ferrocene electrolyte solution were scanned at a scan rate of 0.1 mV s^−1^.

## Results and Discussion

Hexabenzocoronene is carbon–carbon material combined with significant π–π conjugate chemical bonding. A procedure for the preparation for hexabenzocoronene consisted of a series of reactions, such as Sonogashira, Diels-Alder reaction, Lewis catalyst-based cycle reaction, and deprotonation under basic conditions to give intermediates in unsatisfactory yields [36–38]. The target compounds are generated from intermediates and nitromethane with the treatment of a Lewis reagent gave the target compounds in a similarly low yield [39, 40]. The reaction solution was quenched with methanol, followed by repetitive dissolution and precipitation with methylene chloride/methanol. The collected crude compounds were washed with methanol/acetone (1:1) to give a yellowish solid (see Additional file 1) [41, 42].

HBC has been widely used, but in the study of self-assembled system, it needs to be further understood. Although studies of the same or similar anode materials have been mentioned in the reported literature, the HBC study is still insufficient. Therefore, the focus of the work is on the detailed research of the self-assembly system, and put it on one by one to understand the internal dynamic distribution of aggregation and the induction and to improve the supplement of the lack of content anode materials.

**Fig. 2:**
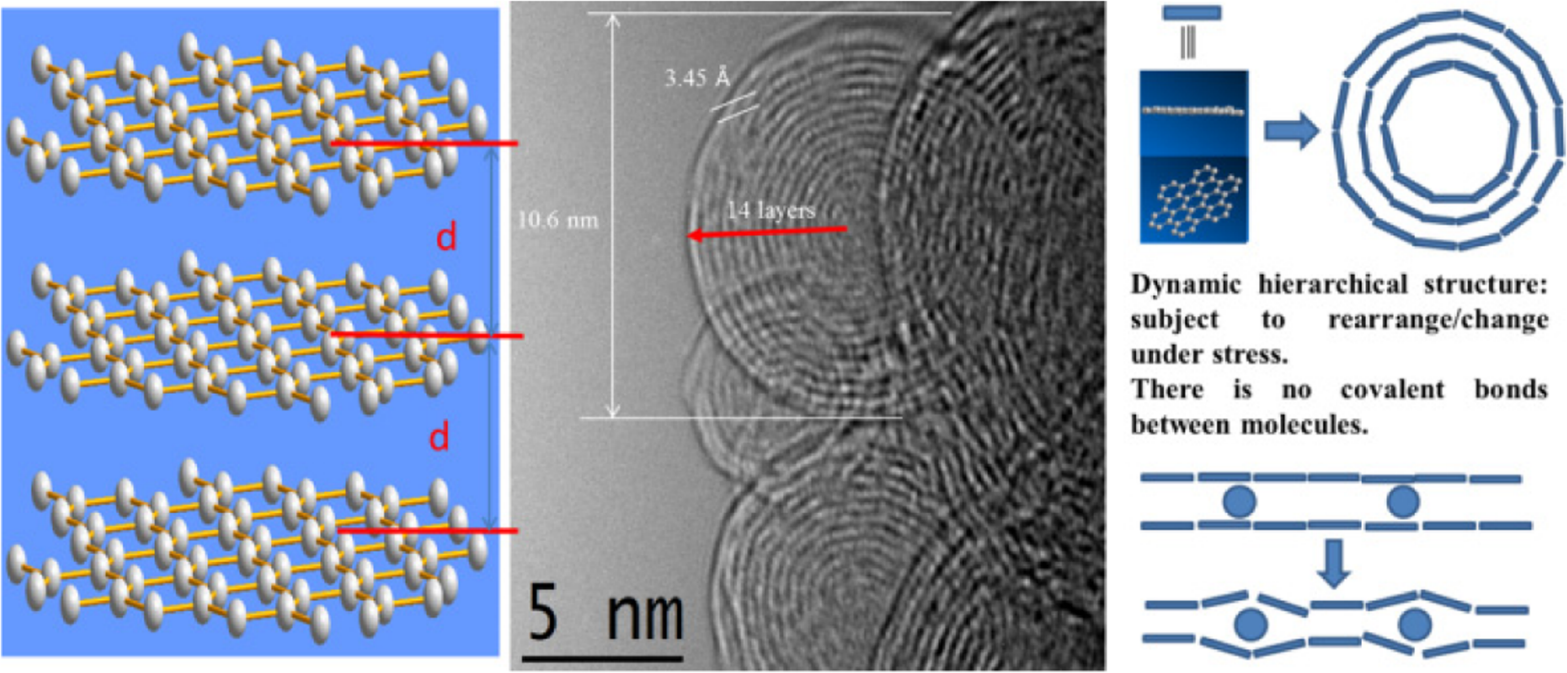
Nano-graphene dynamic hierarchical assemble to rearrange and change

The small-molecule nano-graphene self-assembled dynamically to form regular thin sheets, which were sequentially and systematically stacked to form intermittent sheet nano-graphene fragments that were held tightly to each other [43]. On the other hand, the dynamic self-assembled aggregate structure was superimposed on the subject to rearrange/change under stress, thereby forming an uneven gear shape [44, 45]. Owing to the size of the nano-graphene itself, there was no obvious bulge in the overall structure. As shown in the figure, the entire nano-aggregation was regular, like a fingerprint shape (Fig. 2).

**Fig. 3:**
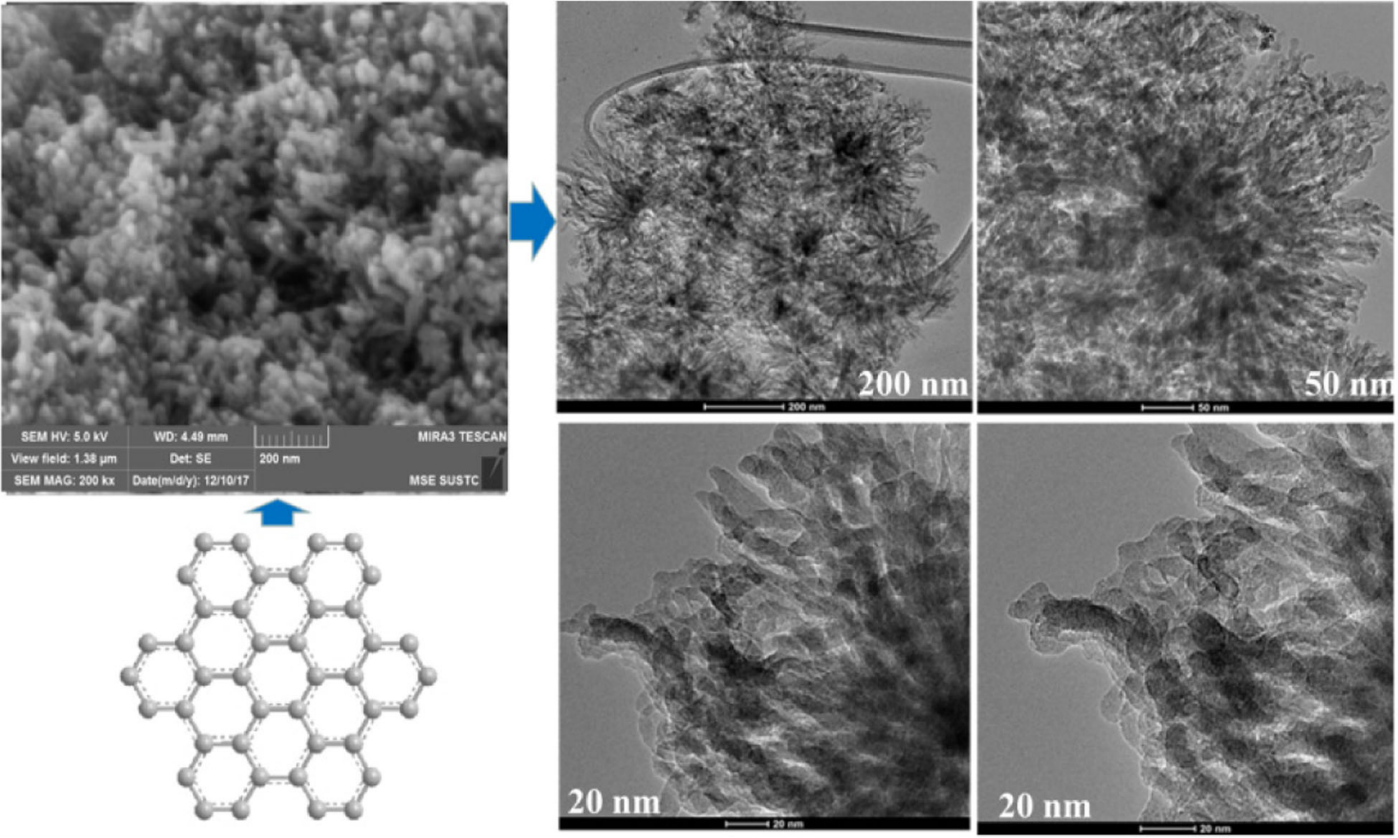
SEM and TEM images for hexabenzocoronene

To explain the abovementioned rearrangement/change caused by its own weight and whether it will affect the material properties, scanning electron microscopy (SEM) was performed to determine if the particle size had changed. As shown in Fig. 3, the nanoparticles are gathered together and their particle size was unaffected by the rearrangement/change. The SEM image clearly shows that nano-graphene was distributed uniformly as nanoparticles. In addition, daisy-like clusters, 200, 50, and 20 nm in range, were observed. Their end parts were stretched outward with certain regularity, which is densely concentrated like a flower pattern. Therefore, the self-assembly process of nano-graphene sheets can be carried out in two ways. First, the nano-graphene molecules are self-assembled by overlapping the edges. Second, nano-graphene molecules overlap with each other, which enables the self-assembly of molecules.

**Fig. 4:**
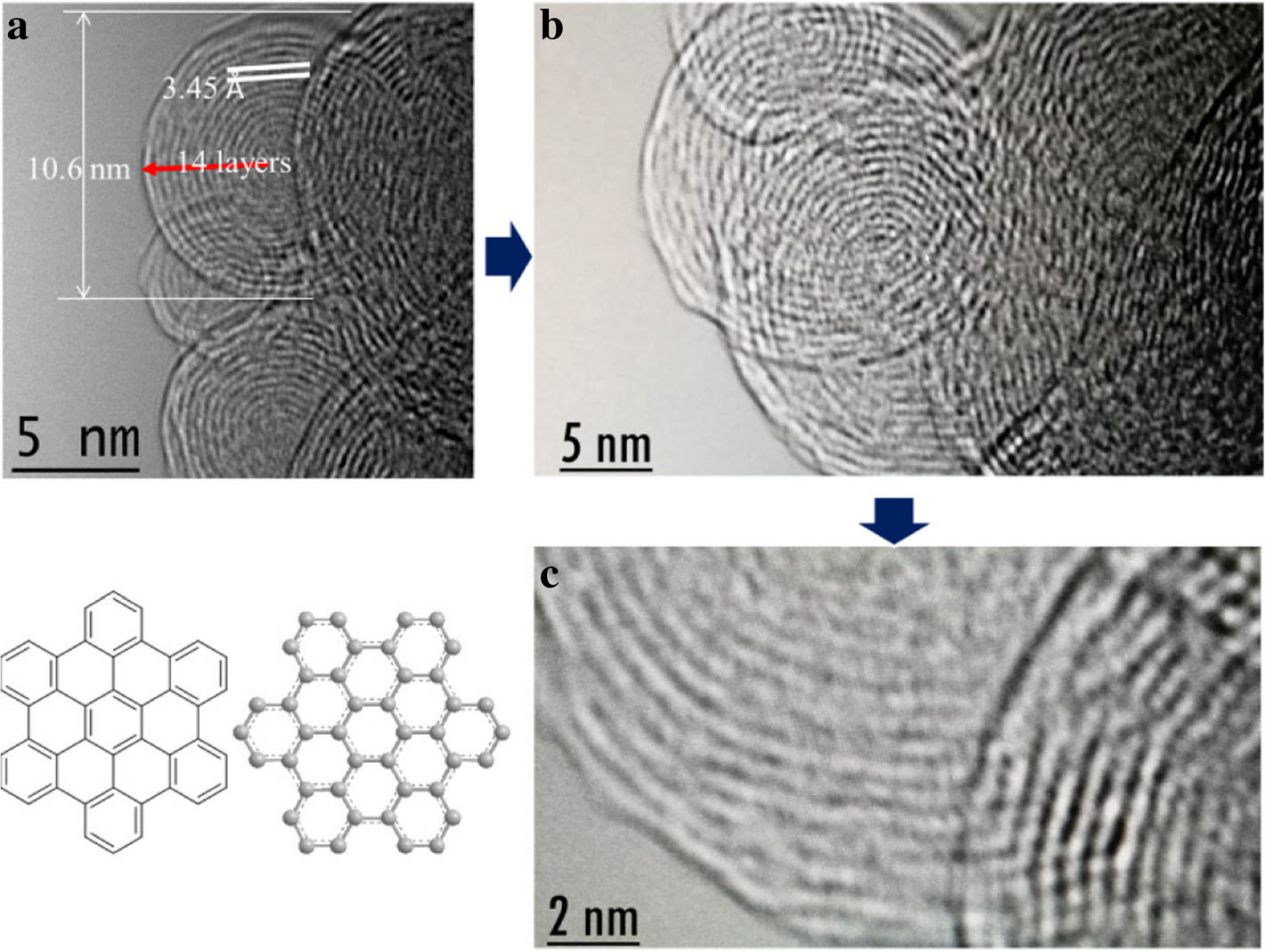
HRTEM image of hexabenzocoronene with their dynamic hierarchical assembles

Transmission electron microscopy (TEM) showed that the hexabenzocoronene molecule exhibits structural features with a coherent layer spacing and a molecular layer spacing from 0.34 nm. High-resolution TEM (HRTEM) indicated that nanoparticles bind to each other (Fig. 4) [46, 47]. The concentric diffraction rings in the selected area electron diffraction (SAED) pattern confirm the polycrystalline nature of hexabenzocoronene. Furthermore, the HRTEM image shows that most of the graphene-like walls consisted of a few layers (≈ 14 layers), indicating typically ultrathin structures [48–51]. The layer-by-layer structures of the hexabenzocoronene and the perfect *d*-spacing between the layers highlight the performance of LIB anode materials.

**Fig. 5:**
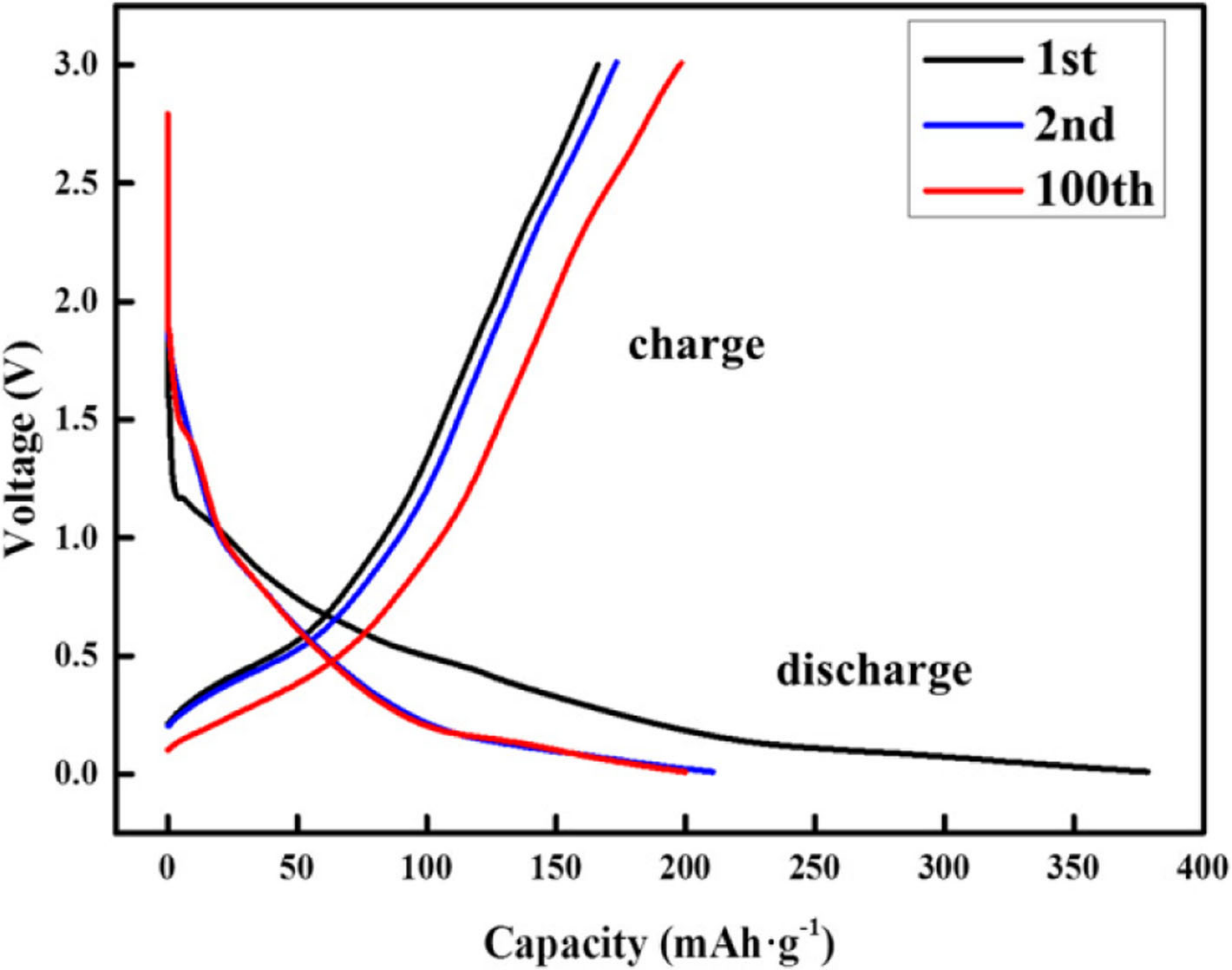
The galvanostatic discharge–charge voltage profiles of hexabenzocoronene anode as a function of cycling numbers

The voltage profiles of hexabenzocoronene and the performance were measured using a cycling test. Figure 5 shows the capacity of the electrode at various current densities and the corresponding voltage profiles. The capacity at 100 cycles is 200 mAh/g, and good reversibility was observed with a coulombic efficiency over 98%.

**Fig. 6:**
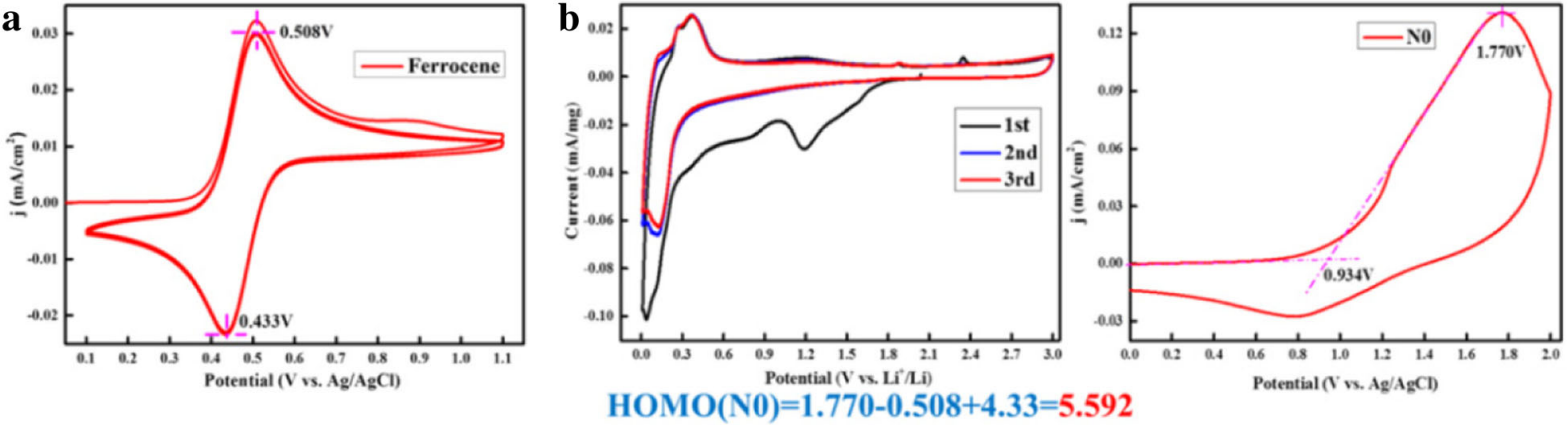
Cyclic voltammograms (CVs) of ferrocene current collector disc vs. silver metal in the electrolyte (**a**) without additive, and **b** oxidation energy HOMO values in acetonitrile using tetrabutylammonium perchloride as electrolyte

Cycle voltage (CV) was performed at the high potential of the lithium-ion batteries to determine the long-term stability and potential energy (Fig. 6a). According to the above description, CV (Li^+^/Li vs Ag/AgCl) was further undertaken to understand the lithium storage behavior. The CV curves hexabenzocoronene were measured at the same scan rates (0.1 mV s^−1^) and display redox peaks with slight shifts with increasing scan rates, thereby showing a rectangular shape with increasing scan rates, as shown in Fig. 6. The twisted rectangular shape at a fast scan rate may be due to the poor electronic nature of the polycrystalline materials, as proposed by Dunn et al. The measured highest occupied molecular orbital (HOMO) energy at a fixed potential (*V*) can be separated into oxidation increases (*V*_1_), standard oxidation effects (*V*_2_), and standard reduction effects (*V*_3_) (Eq. (1)), which can quantitatively characterize the capacity contribution of each part.

1 $$ \mathrm{HOMO}(V)\kern0.5em =\kern0.5em {V}_1\kern0.5em -\kern0.5em {V}_2\kern0.5em +\kern0.5em {V}_3 $$

The anion/radical anion with an electron donating functional group leads to a homogeneous/uniform electron distribution throughout the flake, which is beneficial for maximizing the number of Li^+^ incorporated into the hexabenzocoronene. The charging process (Li^+^ transfer) in hexabenzocoronene anodes requires stabilization. The calculated stabilization HOMO energy of the hexabenzocoronene radical anode ranges from 5.592 V, as shown in Fig. 6b.

**Fig. 7:**
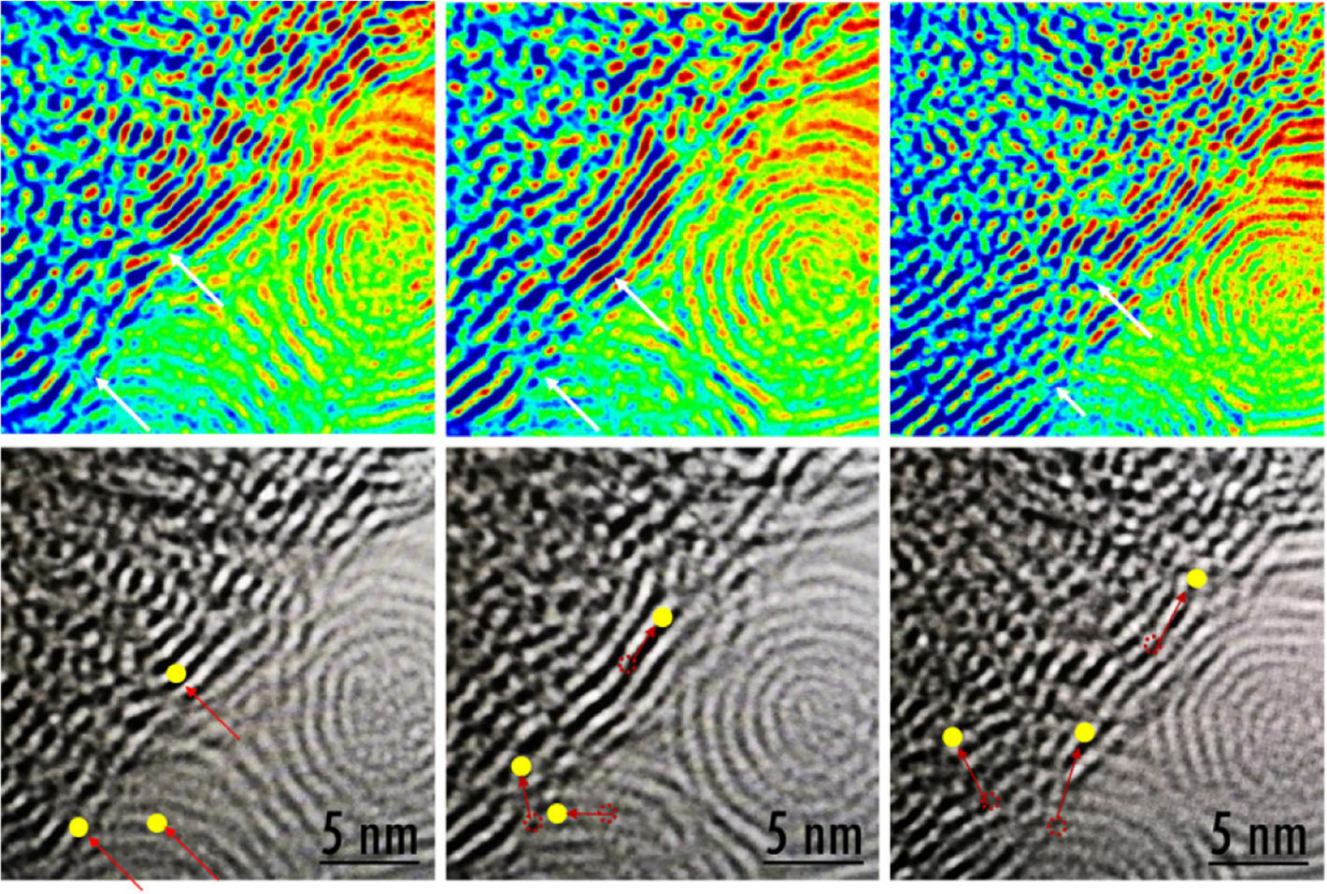
TEM image of nano-graphene multi-stage self-assembly structure

The inset in Fig. 7 shows that the assembled multi-structures experienced arranged and rearranged processes. The optimal *d*-spacing between the layers for hexabenzocoronene was examined. This paper revealed a multi-diffusing process of lithium ions as the dynamic structure providing dynamic diffusion paths. TEM showed that lithium diffuses between the layers and has the ability to pass through the sheets, which greatly increases the lithium ion (yellow spot) diffusion efficiency; the Additional file 1: Figure S1 and Table S1 show adsorption and desorption: *V*_*a*_/cm^3^ (STP) g^−1^ value is 110.47 and 96.62. According to adsorption-desorption isotherm, there is no hysteresis loop in the isotherms of HBC. Moreover, Additional file 1: Figure S2 and Table S2 show BET surface area, and the correlation coefficient value is 0.9999, *V*_*m*_ is 18.647 cm^3^ (STP) g^−1^, and *a*_s,BET_ is 81.16 m^2^ g^−1^. The TEM image revealed self-assembled structures that were disorganized in the center of the fingerprint, and then they were arranged more regularly into a fingerprint-like structure. In the process of the self-assembly of graphene sheets, graphene sheets arrange in a stacked manner and self-assemble into a layered two-dimensional structure in a head-to-head manner. Moreover, the bonding force between molecules is weak with no strong chemical bonds. The self-assembled structure is a dynamic process involving the angular-rearrangement of self-assembled layers of graphene nanosheets under the action of energy. Moreover, TEM image showed that lithium ions have different diffusion modes between the graphene sheets, which can diffuse between the layers and pass through the layers, from the inner layer to the outer layer diffusion. Therefore, nano-graphene exhibits strong lithium ion diffusion properties and surprising lithium ion storage capacity.

## Conclusion

HBC shows good structure durability and stability. The electron density with the optimal *d*-spacing in the self-assemblies led to a significantly enhanced LIB anode charge capacity and cycling stability. These results revealed a structure–property correlation between the nature of functional groups and Li storage capacity. Nevertheless, identifying the mechanism for how nano-graphene hierarchically assembles and dominates the overall battery performance will be an important research topic. Through these studies, a more rational and effective application of nano-graphene will be realized. Observing the characteristics of the internal architecture from a microscopic perspective and analyzing the dynamic hierarchical self-assembly properties of a nano-graphene sheet one by one will be the subjects of a future study.

## Additional File

Additional file 1: Supporting information (DOCX 436 kb)

## Abbreviations

CV: Cycle voltage
HBC: Hexabenzocoronene
HOMO: Highest occupied molecular orbital
HRTEM: High-resolution transmission electron microscope
SAED: Selected area electron diffraction
SEM: Scanning electron microscope
TBAP: Tetra-*n*-butylammonium perchlorate
TEM: Transmission electron microscope
TLC: Thin-layer chromatography
