# Supplementary material for: Dynamic Hierarchical Self-Assemble Small Molecule Structure Hexabenzocoronene for the High-Performance Anodes Lithium Ion Storage
Source: Nanoscale Res Lett. 2019 Feb 26;14:65. doi: 10.1186/s11671-019-2903-4 (PMC6391511; doi:10.1186/s11671-019-2903-4)
Supplement: Supplementary file 1 — Supporting information (DOCX 436 kb) [file 11671_2019_2903_MOESM1_ESM.docx]

Supporting information

Dynamic hierarchical self-assemble small molecule structure hexabenzocoronene for the high performance anodes Lithium ion storage

Dawei He^1^, Fuyan Xiao^3^, Zhou Wang^2^, Aolin He^1^, Ruijiang Liu^3^, and Guofan Jin^3*^

^1^Affiliated Kunshan Hospital, Jiangsu University, Kunshan 215300, P.R. China

^2^College of Vanadium and Titanium, Panzhihua University, 617000, P.R. China

^3^School of Pharmacy, Jiangsu University, Zhenjiang, 212013, P. R. China

*Corresponding authors: [organicboron@ujs.edu.cn](mailto:organicboron@ujs.edu.cn) (G. F. Jin).

**General Information on Techniques**

Mass spectra were measured on a Waters Xevo OTof MS with an ASAP probe. Electrospray ionization (ESI) mass spectra were recorded on a Thermoquest Trace or a MALDI-TOF-Mass. Elemental analyses were performed using a Carlo Erba Instruments CHNS-O EA1108 analyzer.

- 1. **Materials synthesis**

1,2-diphenylethyne^[1]^ and 2,3,4,5-tetraphenylcyclopenta-2,4-dien-1-one^[2]^,were synthesized according to a previously reported procedure. All other reagents were used as received from commercial sources.

- 1. General Procedure for oxidation cyclization reaction

Compound 3',4',5',6'-tetraphenyl-1,1':2',1''-terphenyl (0.23g, 0.43mmol) was dissolved in dry dichloromethane (5 mL). The solution was degassed via bubbling nitrogen for 10min. Then FeCl3 (1.4g, 8.6mmol) in dry nitromethane (5 mL) was added slowly via a syringe. The resulting mixture was kept under nitrogen flow during the entire reaction. After 5 h, the reaction solution was quenched with methanol, followed by repetitive dissolution and precipitation with methylene chloride / methanol. The collected crude compounds were washed with methanol / acetone (1:1). The final yellowish precipitate was then collected and dried in a vacuum to afford hexabenzocoronene Yield: 0.08 g (36%). ESI–MS (M + H)^+^: 522.48 for Calculated 522.50; Found, %: C, 96.53; H, 3.47. C42H18. Calculated, %: C, 96.71; H, 3.42.

**

**

Tabel S1. Measurement of hexabenzocoronene adsorption / desorption isotherm data.

| No | *pi*/kPa | *pe*/kPa | *pe*2/kPa | *p*_0_/kPa | *p*/*p*_0_ | *V_a_*/cm^3^(STP) g^-1^ |
| --- | --- | --- | --- | --- | --- | --- |
| ADS |  |  |  |  |  |  |
| 1 | 0 | 0.00015336 | 0 | 106.54 | 1.4395E-06 | 0.1286 |
| 2 | 0 | 0.0017882 | 0 | 106.47 | 0.000016795 | 0.3456 |
| 3 | 0 | 0.0031816 | 0 | 104.65 | 0.000030402 | 0.4304 |
| 4 | 0 | 0.0097022 | 0 | 104.55 | 0.0000928 | 0.6298 |
| 5 | 0 | 0.4737 | 0 | 104.58 | 0.0045294 | 2.5585 |
| 6 | 0 | 0.8266 | 0 | 104.61 | 0.0079017 | 3.138 |
| 7 | 0 | 1.1725 | 0 | 104.59 | 0.01121 | 3.2245 |
| 8 | 0 | 4.9498 | 0 | 104.62 | 0.047312 | 6.985 |
| 9 | 0 | 8.2127 | 0 | 104.63 | 0.078493 | 9.1228 |
| 10 | 0 | 11.524 | 0 | 104.65 | 0.1101 | 11.249 |
| 11 | 0 | 17.422 | 0 | 104.64 | 0.1665 | 14.533 |
| 12 | 0 | 22.849 | 0 | 104.62 | 0.2184 | 17.265 |
| 13 | 0 | 28.353 | 0 | 104.63 | 0.271 | 19.894 |
| 14 | 0 | 33.843 | 0 | 104.61 | 0.3235 | 22.815 |
| 15 | 0 | 37.383 | 0 | 104.62 | 0.3573 | 24.314 |
| 16 | 0 | 42.956 | 0 | 104.62 | 0.4106 | 26.309 |
| 17 | 0 | 48.152 | 0 | 104.61 | 0.4603 | 28.421 |
| 18 | 0 | 53.406 | 0 | 104.57 | 0.5107 | 30.588 |
| 19 | 0 | 58.646 | 0 | 104.55 | 0.5609 | 32.499 |
| 20 | 0 | 63.877 | 0 | 104.52 | 0.6111 | 34.161 |
| 21 | 0 | 69.095 | 0 | 104.5 | 0.6612 | 36.169 |
| 22 | 0 | 74.311 | 0 | 104.45 | 0.7115 | 39.52 |
| 23 | 0 | 79.483 | 0 | 104.4 | 0.7613 | 42.436 |
| 24 | 0 | 84.608 | 0 | 104.37 | 0.8107 | 46.348 |
| 25 | 0 | 89.697 | 0 | 104.33 | 0.8597 | 52.795 |
| 26 | 0 | 94.595 | 0 | 104.31 | 0.9069 | 63.889 |
| 27 | 0 | 99.357 | 0 | 104.28 | 0.9528 | 83.636 |
| 28 | 0 | 101.48 | 0 | 104.25 | 0.9734 | 93.541 |
| 29 | 0 | 103.22 | 0 | 103.8 | 0.9944 | 110.47 |
| DES |  |  |  |  |  |  |
| 1 | 0 | 100.63 | 0 | 103.78 | 0.9696 | 96.62 |
| 2 | 0 | 98.052 | 0 | 103.79 | 0.9447 | 88.382 |
| 3 | 0 | 93.131 | 0 | 103.84 | 0.8969 | 74.142 |
| 4 | 0 | 87.921 | 0 | 103.84 | 0.8467 | 58.666 |
| 5 | 0 | 82.608 | 0 | 103.82 | 0.7957 | 47.312 |
| 6 | 0 | 77.33 | 0 | 103.78 | 0.7451 | 39.273 |
| 7 | 0 | 72.078 | 0 | 103.75 | 0.6947 | 33.247 |
| 8 | 0 | 66.864 | 0 | 103.69 | 0.6448 | 28.865 |
| 9 | 0 | 61.661 | 0 | 103.65 | 0.5949 | 25.109 |
| 10 | 0 | 56.482 | 0 | 103.64 | 0.545 | 22.267 |
| 11 | 0 | 51.329 | 0 | 103.64 | 0.4953 | 19.715 |
| 12 | 0 | 46.149 | 0 | 103.63 | 0.4453 | 16.991 |
| 13 | 0 | 40.966 | 0 | 103.59 | 0.3955 | 15.337 |
| 14 | 0 | 35.807 | 0 | 103.56 | 0.3458 | 14.003 |
| 15 | 0 | 30.657 | 0 | 103.53 | 0.2961 | 11.96 |
| 16 | 0 | 24.098 | 0 | 103.51 | 0.2328 | 10.413 |
| 17 | 0 | 20.659 | 0 | 103.49 | 0.1996 | 9.5775 |
| 18 | 0 | 14.279 | 0 | 103.49 | 0.138 | 7.7984 |
| 19 | 0 | 10.832 | 0 | 103.47 | 0.1047 | 6.9649 |
| 20 | 0 | 7.3893 | 0 | 103.44 | 0.071436 | 5.8551 |
| 21 | 0 | 3.9251 | 0 | 103.41 | 0.037957 | 5.0893 |
| 22 | 0 | 1.0447 | 0 | 103.42 | 0.010102 | 3.4266 |
| 23 | 0 | 0.6306 | 0 | 103.4 | 0.0060985 | 2.9447 |
| 24 | 0 | 0.3465 | 0 | 103.4 | 0.0033509 | 2.4855 |
| 25 | 0 | 0.1161 | 0 | 103.39 | 0.0011225 | 1.8217 |
| 26 | 0 | 0.096592 | 0 | 103.38 | 0.00093434 | 1.7249 |


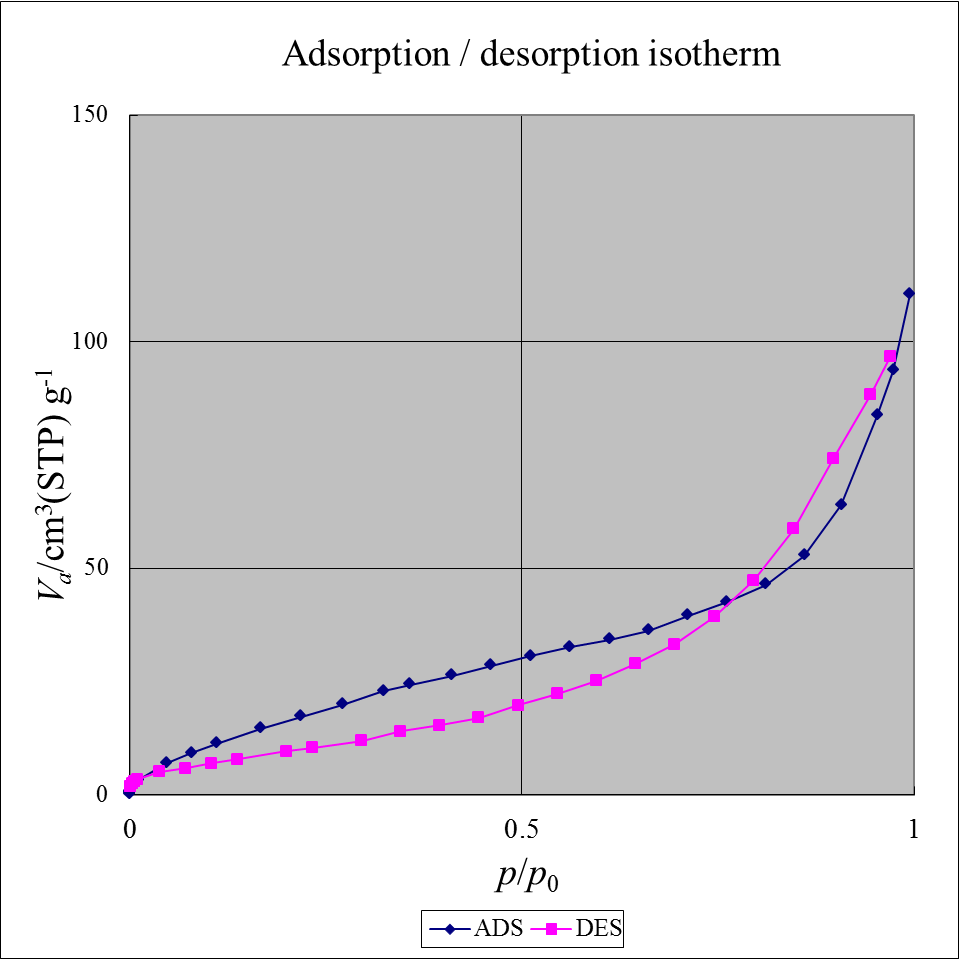


Figure S1. Hexabenzocoronene of adsorption / desorption isotherm graph.

Tabel S2. Measurement of hexabenzocoronene BET plot data.

| Starting point |  |  | 10 |  |  |
| --- | --- | --- | --- | --- | --- |
| End point |  |  | 12 |  |  |
| Slope(Linear) |  |  | 0.047886 |  |  |
| Intercept(Linear) |  |  | 0.0057418 |  |  |
| Correlation coefficient | |  | 0.9999 |  |  |
| *V_m_* |  |  | 18.647 | [cm^3^(STP) g^-1^] |  |
| a_s,BET_ |  |  | 81.16 | [m^2^ g^-1^] |  |
| *C* |  |  | 9.3399 |  |  |
| Total pore volume(*p*/*p*_0_=0.990) | |  | 0.1654 | [cm^3^ g^-1^] |  |
| Mean pore diameter | |  | 8.1501 | [nm] |  |

| No | *p*/*p*_0_ | *p*/*V_a_*(*p*_0_-*p*) |
| --- | --- | --- |
| 0 | 0 | 0 |
| 1 | 1.4395E-06 | 0.000011189 |
| 2 | 0.000016795 | 0.000048591 |
| 3 | 0.000030402 | 0.000070644 |
| 4 | 0.0000928 | 0.00014736 |
| 5 | 0.0045294 | 0.0017784 |
| 6 | 0.0079017 | 0.0025381 |
| 7 | 0.01121 | 0.0035161 |
| 8 | 0.047312 | 0.0071098 |
| 9 | 0.078493 | 0.0093369 |
| 10 | 0.1101 | 0.011001 |
| 11 | 0.1665 | 0.013745 |
| 12 | 0.2184 | 0.016185 |
| 13 | 0.271 | 0.018685 |
| 14 | 0.3235 | 0.020961 |
| 15 | 0.3573 | 0.022867 |
| 16 | 0.4106 | 0.026478 |
| 17 | 0.4603 | 0.030009 |


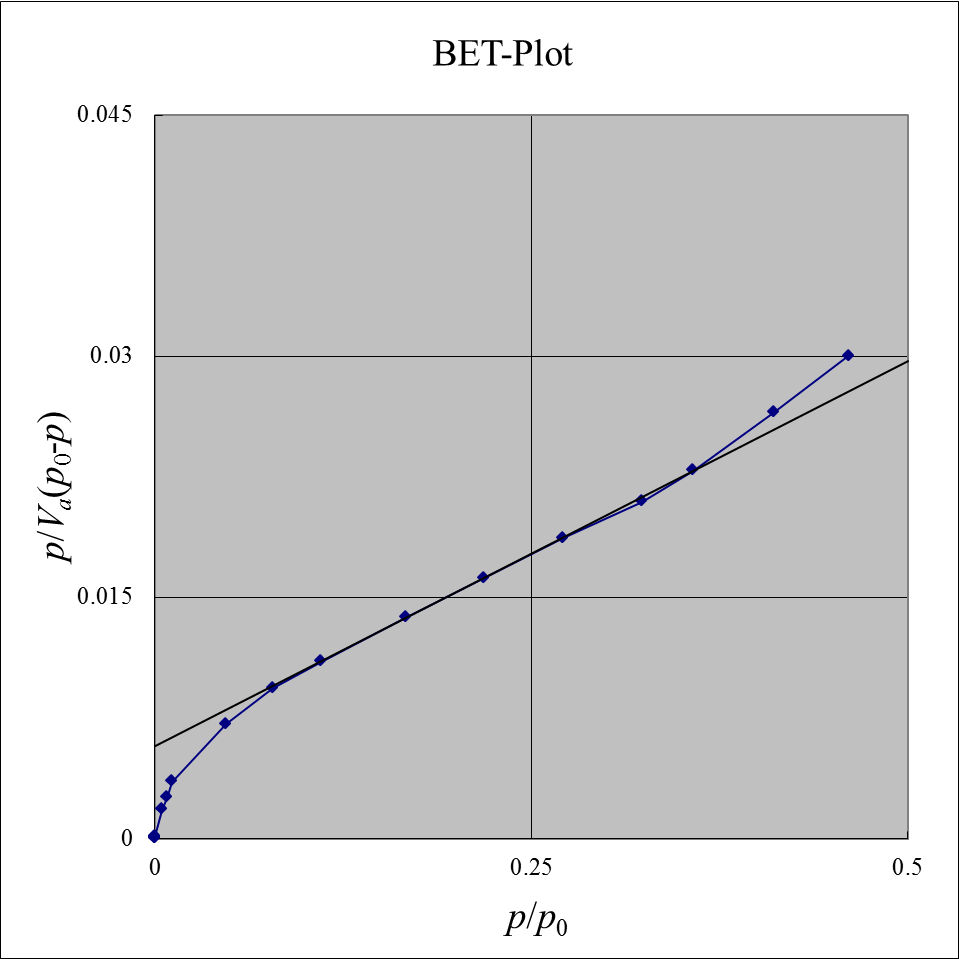


Figure S2. Hexabenzocoronene of BET-Plot graph.

References

[1].Cope, A. C.; Smith, D. S.; Cotter, R. J. [*Organic Syntheses*](https://en.wikipedia.org/wiki/Organic_Syntheses). 1963, 4, 377*-*380.

[2].John R. Johnson, J. R.; Grummitt, O. [*Organic Syntheses*](https://en.wikipedia.org/wiki/Organic_Syntheses). 1943, 23, 92-93.
